# Feasibility of Using the Apple Vision Pro for Diagnostic Radiology: User Experience and Perceived Clinical Utility

**DOI:** 10.1007/s10278-025-01745-7

**Published:** 2025-11-17

**Authors:** Joshua Volin, Vidya Viswanathan, Elizabeth A. Krupinski, Nabile Safdar, Rachel Silverman, Vikram Narayan, Elias Kikano

**Affiliations:** 1 Department of Radiology and Imaging Science, Emory University School of Medicine, 1364 Clifton Rd NE, Atlanta, GA 30322 USA; 2 Department of Urology, Emory University School of Medicine, 1364 Clifton Rd NE, Atlanta, GA 30322 USA; 3 Emory Healthcare, 1364 Clifton Rd NE, Atlanta, GA 30322 USA

**Keywords:** Radiology, Education, Spatial computing, Augmented reality, Anatomy, AR/VR

## Abstract

To assess the feasibility and user experience of spatial computing with Apple Vision Pro (AVP) in medical imaging radiology education. This prospective observational pilot study, approved by the Institutional Review Board and HIPAA-compliant, was conducted at a multisite academic medical center from January to March 2025. Twenty-five radiology physicians (22 diagnostic radiology residents, 2 neuroradiology fellows, 1 neuroradiology attending) participated. After a 5-min setup, participants engaged in a combined structured and self-guided trial of the AVP for clinical applications such as image viewing and electronic health record navigation. Participants completed pre-use and post-use surveys to assess user familiarity, comfort, and perceived utility using 5-point Likert scales. Matched responses were analyzed with Wilcoxon signed-rank tests. Timing data for onboarding and total session duration were recorded. Mean time to device competency was 4.0 ± 1.37 min, and average session time was 14.1 ± 4.94 min. Significant pre-to-post improvements were observed in perceived utility for multidisciplinary conferences (*p* = 0.039). The majority rated overall experience and ease of use ≥ 4/5 post-use. Eye strain occurred in 12%; no users reported nausea. Most participants cited ease of use (84%) and efficiency (80%) as key factors influencing future adoption. In this small-scale pilot, the AVP demonstrated strong short-term feasibility and perceived usability for medical imaging among surveyed physician trainees. Results support further investigation of spatial computing in other clinical workflows and medical imaging education.

**Supplementary Information:**

The online version contains supplementary material available at https://doi.org/10.1007/s10278-025-01745-7.

## Introduction

Radiology education has traditionally relied on didactic lectures, case-based learning, and hands-on experience with imaging modalities on conventional 2D workstations [1]. In recent years, there has been a shift toward interactive, technology-driven methods such as simulation-based training, artificial intelligence-assisted learning, and extended reality (XR) technologies, including augmented reality (AR) and virtual reality (VR) headsets. These tools enhance understanding of anatomy, improve recall, and provide risk-free procedural training environments, demonstrating improved learner engagement and short-term knowledge retention compared to traditional approaches [2–6]. They also enable exploration of 3D models derived from patient imaging, virtual reading rooms, and simulation of image-guided interventions [7, 8].

Despite these advantages, prior generations of AR/VR systems have faced notable limitations, including low display resolution, latency, and insufficient integration with clinical imaging systems [9, 10]. Early studies in medical extended reality (MXR) reported educational benefits, but widespread adoption remained limited due to high cost, bulky ergonomics, and technical barriers such as inconsistent image rendering and lack of DICOM calibration [2, 11, 12]. Consequently, these devices are often confined to research or demonstration settings rather than integrated into clinical or educational workflows.

The introduction of the Apple Vision Pro (AVP) (Apple Inc., Cupertino, CA) on February 2, 2024, marked a significant advancement in hardware and introduction to spatial computing. The device combines previous AR and VR technologies through high-resolution micro-OLED displays (approximately 4 K per eye; 3660 × 3200 pixels) that deliver exceptional luminance, contrast, and spatial fidelity, enabling visualization of fine anatomical details critical for diagnostic interpretation [11, 13]. In addition, its self-contained hardware design, eye- and hand-tracking navigation, and integration with a broader software ecosystem distinguish it from earlier-generation systems. Recent studies have demonstrated feasibility across multiple healthcare domains, including use as an immersive operative display in minimally invasive surgery and for improved targeting accuracy and reduced mental strain in hepatic ablation procedures [14–16].

Despite these advances, AR/VR integration into diagnostic imaging remains rare, limited by the lack of large-scale validation and uncertainty regarding ergonomic feasibility and clinical utility [9, 10, 17]. Continued evaluation is needed to clarify how spatial computing platforms may contribute to medical imaging and education.

The aim of this study was to evaluate the perceived feasibility of integrating spatial computing and the Apple Vision Pro into medical imaging education and radiology workflows, to assess user experience across various levels of training, and to explore the perceived value of this device for diagnostic image visualization, informatics interaction, and immersive 3D medical education. By examining these domains, this study seeks to clarify the potential role of spatial computing in shaping future radiology education and practice.

## Methods

### Study Setting and Design

This was a prospective observational pilot study conducted at a multisite academic medical center in the United States. Data collection occurred between January and March 2025. Participants provided informed consent prior to enrollment. The study was approved by the Institutional Review Board and complied with the Health Insurance Portability and Accountability Act (HIPAA).

### Study Participants

The study enrolled 22 radiology residents (21 diagnostic, 1 nuclear medicine), 2 neuroradiology fellows, and 1 neuroradiology attending. Participants were aged 20–60 years, with most (60%) between ages 30–45. Eligible participants were radiology physicians-in-training or practicing faculty. Exclusion criteria included a prior history of seizure disorders, severe motion sickness, or visual acuity deficits requiring corrective lens (soft contact lens were permitted for use).

### Data Sources and Collection

**Fig. 1:**
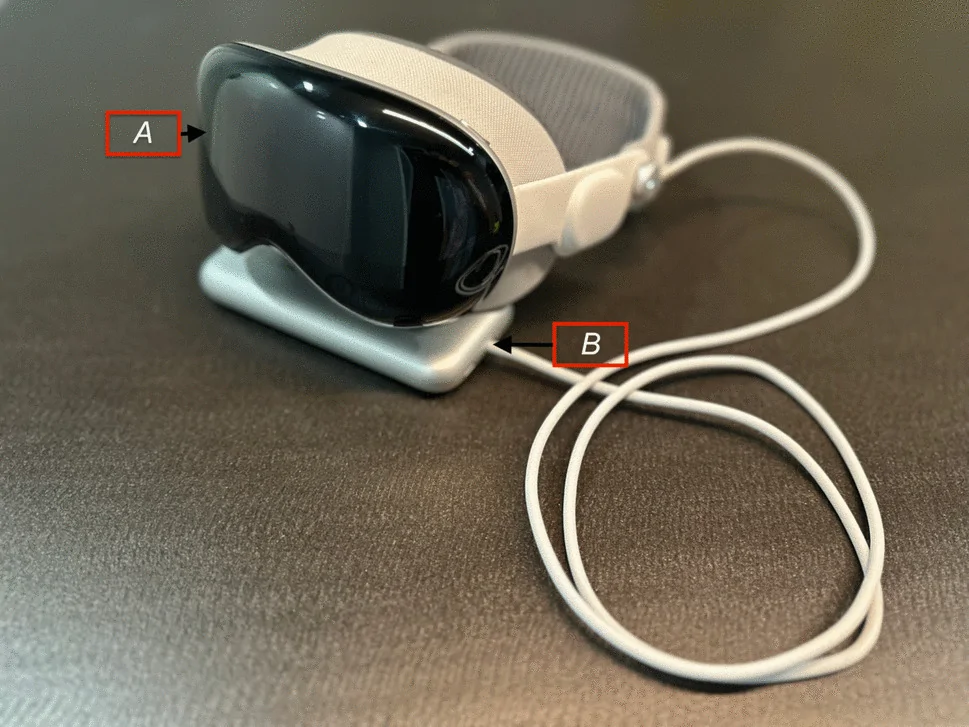
Apple vision pro head set and components. 1**A** – headset, 1**B** – battery

Survey instruments were developed to assess user demographics, prior familiarity with VR, and initial perceptions regarding clinical applications of AR/VR technology. Pre-use surveys assessed baseline knowledge and expectations, and post-use surveys evaluated usability, comfort, and perceived utility of spatial computing with AVP (Appendix A). Likert scales (1 = strongly disagree/low, 5 = strongly agree/high) were used throughout. Participants completed onboarding and interacted with Picture Archiving and Communications Systems (PACS) and Electronic Health Record (EHR) applications running natively on the AVP (Fig. 1).

Timing data, including minutes to achieve self-assessed competency and total session duration, were collected manually by trained observers (Appendix B). Data was entered in real-time to ensure accuracy.

### Intervention

Participants first scanned a QR code that directed them to the pre-intervention survey. Upon completion of the survey, they were provided the AVP headset running visionOS 2.3 in guest mode. The study leader recorded this moment as the start time. Participants then completed the guest mode calibration sequence.

Following device calibration, participants were asked whether they felt comfortable using the device. Once they verbally confirmed their comfort, the study leader marked the time to self-assessed competency. Participants were encouraged to freely explore non-clinical applications and AVP settings to become familiar with the user interface and navigation environment. During this period, they were allowed to ask questions, and the study leader provided verbal prompts to encourage exploration of specific functionalities.

**Fig. 2:**
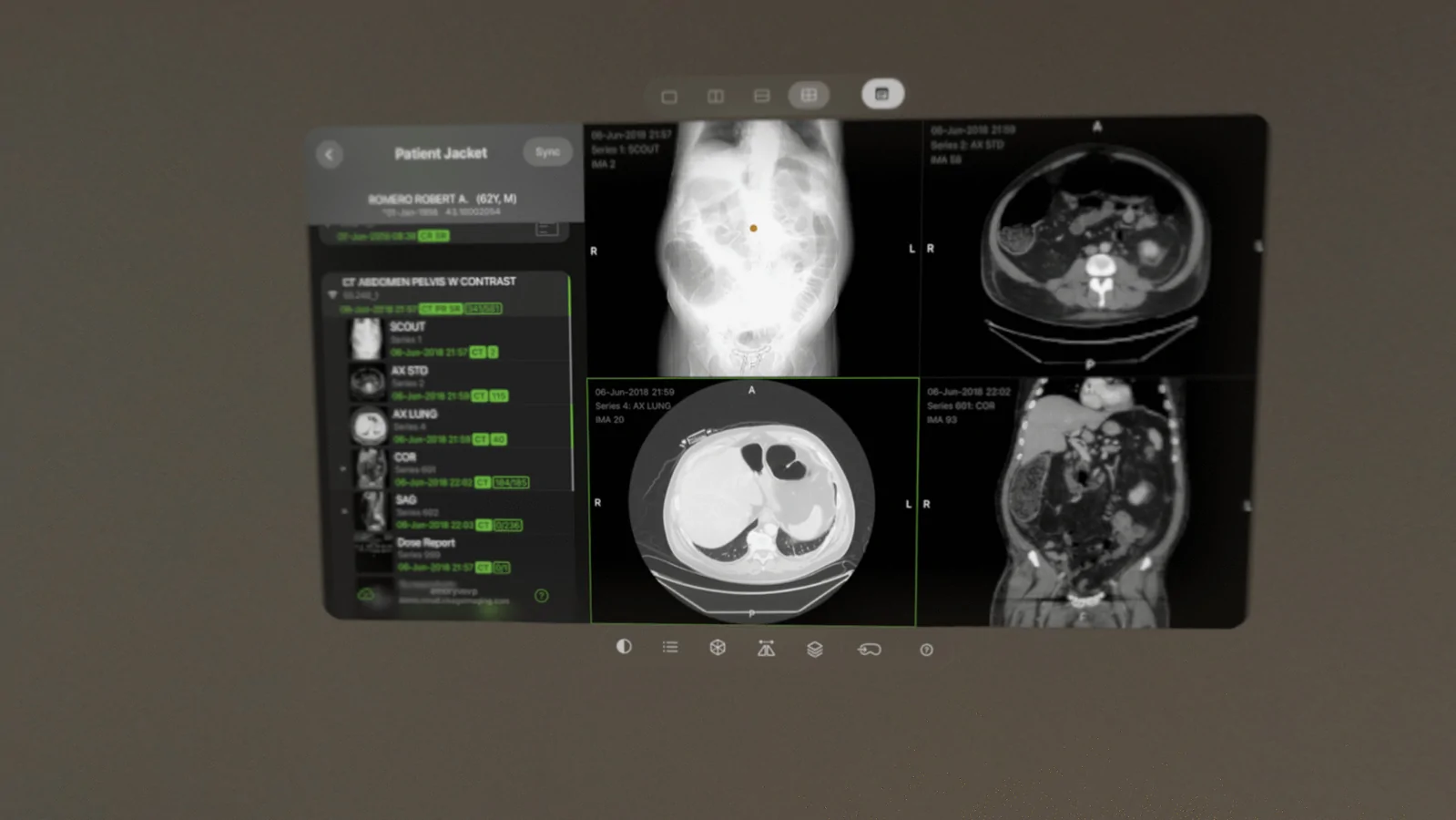
Visage ease VP for PACS simulation

**Fig. 3:**
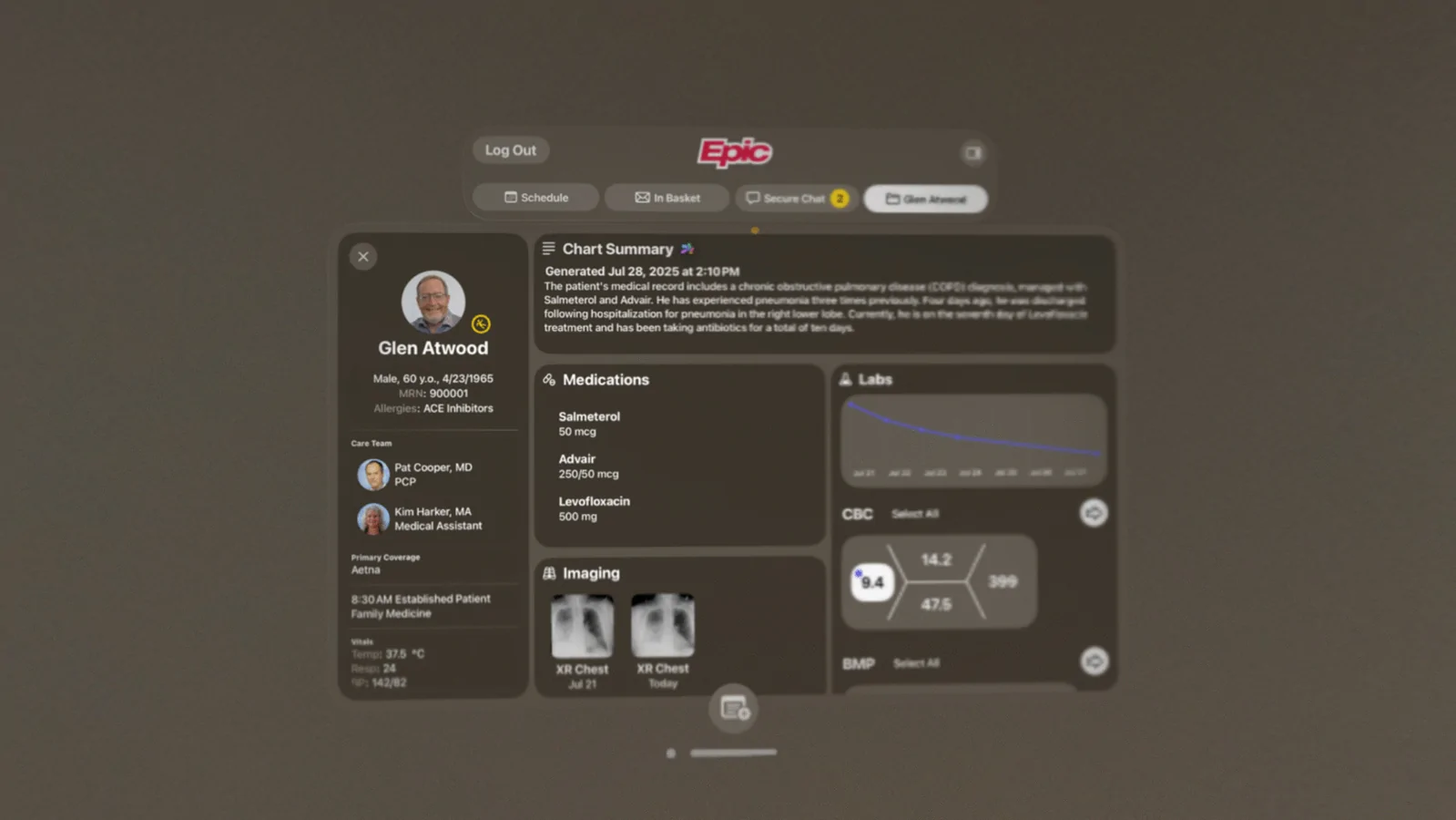
Epic spectacles for EMR Interaction (Fictious Patient)

**Fig. 4:**
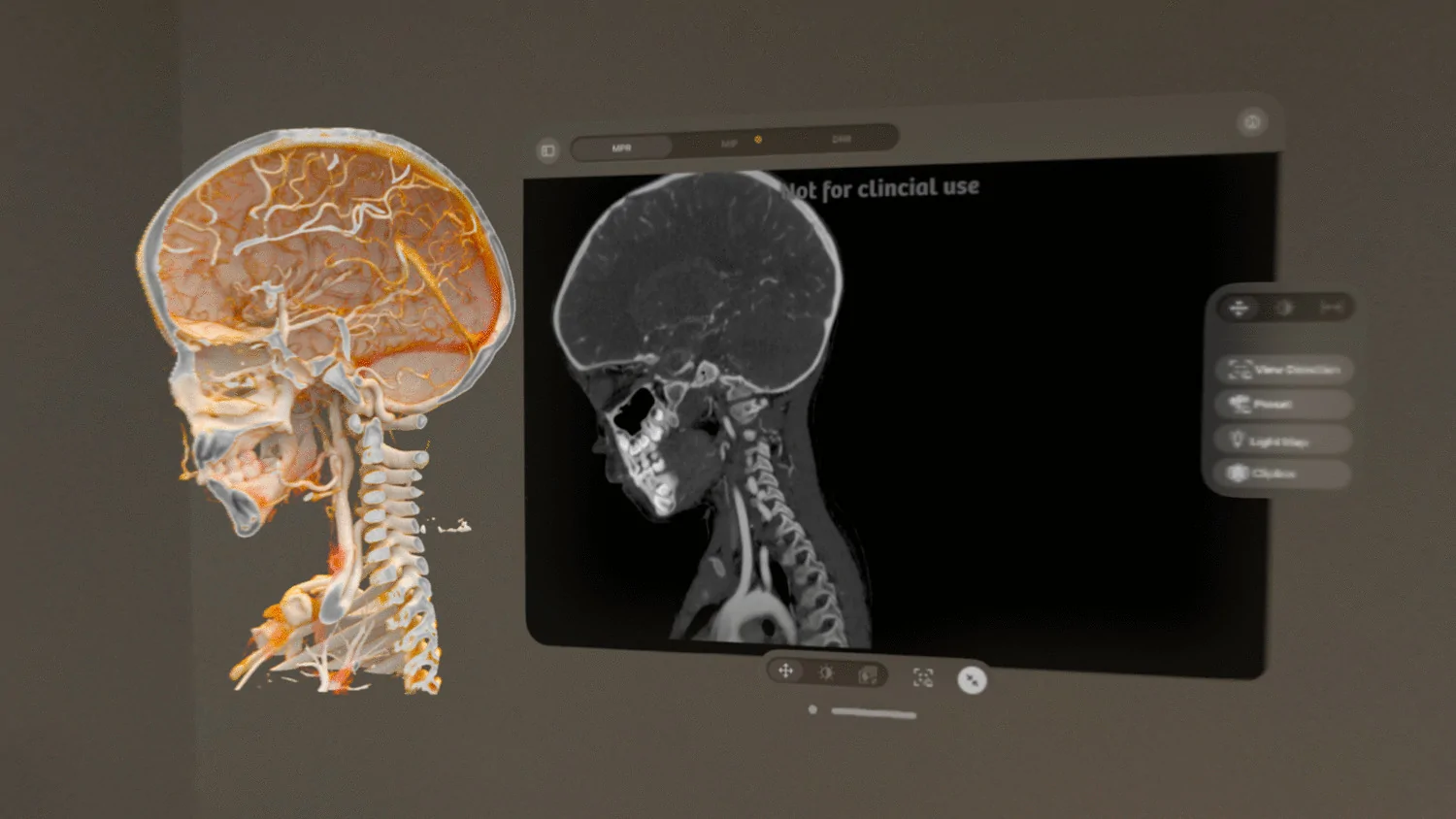
Siemens cinematic reality for 3-D rendering

When the participant indicated that they were comfortable with AVP and self declared that they were done exploring non-clinical apps, the study leader directed them to launch the clinical applications pre-installed on the device. These included Visage Ease VP (Visage Imaging, San Diego, CA) for PACS medical imaging 2D and 3D viewing (Fig. 2), Epic Spectacles (Epic, Madison, WI) for EHR interaction (Fig. 3), and Siemens Cinematic Reality (Siemens, Erlangen, Germany) for additional 3D rendering (Fig. 4).

Participants then conducted a series of tasks in a self-guided manner, including diagnostic image viewing, image manipulation, 3D volume rendering, and electronic health record review. Once participants signaled that they had completed their exploration and were satisfied with the experience, the headset was removed. The study leader marked the intervention end time, and participants were asked to complete the post-intervention survey.

### Outcomes

Primary outcomes included feasibility (onboarding time, device competency, total session duration) and user-rated perceived utility of spatial computing in AVP across clinical and educational domains. Secondary outcomes included changes in outlook from pre- to post-use and usability ratings (e.g., ergonomics, image quality, potential for integration into workflow).

### Statistical Analysis

Matched pre- and post-survey responses were analyzed using the Wilcoxon signed-rank test. Chi-square tests were used to examine associations between user demographics (age group) and categorical survey responses. Descriptive statistics summarized central tendency and dispersion for timing and Likert-based data. Analyses were performed using Microsoft Excel and R (v4.2.1 R Foundation for Statistical Computing, Vienna, Austria).

## Results

### Study Cohort

There were 25 survey respondents with the majority being radiology resident physicians (*n* = 22, 88%; 21 (95%) diagnostic and 1 (5%) nuclear medicine), followed by neuroradiology fellow physicians (*n* = 2, 8%) and a neuroradiology attending physician (*n* = 1, 4%). Given the distribution, training level was not used in further analyses as a variable. The majority were 30–45 years old (*n* = 15, 60%) followed by 20–30 (*n* = 9, 36%) and 45–60 (*n* = 1, 4%). For analyses 30–45 and 45–60 were combined.

More than half (*n* = 15, 60%) play video games with no difference as a function of age group (X^2^ = 0.26, *p* = 0.609). Only 12% (*n* = 3) were very familiar with VR, 44% (*n* = 11) familiar, and 44% (*n* = 11) not familiar with no difference as a function of age group (X^2^ = 0.21, *p* = 0.347). Just over half (*n* = 14, 56%) have used VR with no difference as a function of age group (X^2^ = 0.001, *p* = 0.973).

### Feasibility Metrics

Mean time to competency was 4.0 ± 1.37 min (range: 2–8), and mean total session time was 14.1 ± 4.94 min (range: 6–27).

### Pre- and Post-Use Survey: Perceived Utility

Table 1 shows the pre- and post-use distribution of responses (1–5 scale where 1 = low, 5 = high) to a series of questions asking respondents to “Please rate how likely VR technology has a role in….”. Although all post-use responses tended to be more positive (4 and 5), only a role in multidisciplinary conferences reached significance. Two additional questions asked about image quality and the ability to dictate using a VR headset. Although they also tended to more positive, they did not reach statistical significance.

**Table 1:** Distributions of responses for pre- and post-use surveys on questions regarding “Please rate how likely VR technology has a role in….” (1–5 scale where 1 = low, 5 = high)

	1	2	3	4	5	X^2^ & p-value	Medians	Means
	Pre/post	Pre/post	Pre/post	Pre/post	Pre/post		Pre/Post	Pre/Post
**radiology/imaging**	4%/0%	28%/12%	24%/24%	28%/60%	16%/4%	X^2^ = 16.89 p = 0.154	3.0/3.24	4.0/3.56
**consultations with referring colleagues**	4%/0%	32%/12%	24%/32%	36%/36%	4%/20%	X^2^ = 17.69 p = 0.126	3.0/3.04	4.0/3.64
**interventional radiology**	4%/0%	20%/16%	44%/32%	20%/44%	12%/8%	X^2^ = 16.89 p = 0.154	3.0/3.16	4.0/3.44
**multidisciplinary conferences**	0%/0%	28%/8%	32%/32%	28%/44%	12%/16%	X^2^ = 17.61 p = 0.039	3.0/3.24	4.0/3.44
**medical education**	0%/0%	4%/4%	32%/4%	44%/60%	20%/32%	X^2^ = 9.19 p = 0.420	4.0/3.8	4.0/4.2
**simulation training**	0%/0%	12%/4%	16%/4%	44%/48%	28%/44%	X^2^ = 14.89 p = 0.094	4.0/3.88	4.0/4.32
**clinical care**	4%/0%	32%/21%	44%/25%	20%/37%	0%/17%	X^2^ = 11.23 p = 0.261	3.0/2.8	4.0/3.5
**patient education and therapy**	0%/0%	8%/8%	40%/12%	44%/64%	8%/16%	X^2^ = 12.99 p = 0.163	4.0/3.52	4.0/3.88
**image quality**	0%/0%	20%/4%	36%/12%	32%/56%	12%/28%	X^2^ = 6.40 p = 0.699	3.0/3.36	4.0/4.08
**ability dictate**	0%/0%	28%/4%	12%/32%	24%/40%	36%/24%	X^2^ = 10.36 p = 0.322	4.0/3.68	4.0/3.84

### Pre-Survey: General Outlook

Additional unmatched pre-use questions addressed other topics. In terms of the role of AR in diagnostic imaging, 68% saw it as a tool, 52% as a novelty, 4% as an adjunct tool, and 4% as a replacement with no difference ass a function of age group (X^2^ = 2.31, *p* = 0.511). Potential advantages (could select more than 1 response) were 76% for education, 68% mobility, 48% efficiency, and 40% quality with no differences as a function of age group (X^2^ = 0.67, *p* = 0.881). Disadvantages were noted as slow processing speed (72%) and limited usage (52%) with no difference as a function of age group (X^2^ = 2.43, *p* = 0.119), and there were 3 “other” responses written in (hand movement efficiency, human error, learning efficiency).

With respect to how likely (1–5 scale) they would incorporate new technologies like the AVP into daily practice, 4% rated 1 (unlikely), 36% 2, 24% 3, 16% 4 and 20% 5 (likely) with no difference as a function of age group (X^2^ = 1.76, *p* = 0.781). Primary motivations for participating in the study (could select more than 1 response) were exposure to new technology (92%), general interest (64%), and additions to current practice (36%) with no difference as a function of age group (X^2^ = 1.19, *p* = 0.553). The key factors that would influence the decision to use the AVP regularly in practice were (could select more than 1 response) overall ease of use (84%), improved efficiency (80%), ergonomics (80%), and improved quality of care (60%) with no difference as a function of age group (X^2^ = 1.32, *p* = 0.724).

### Post-Use Usability and Visual Perception

Other unmatched post-use questions (Table 2) used a 5-point scale (where 1 = low, 5 = high) and results are presented in Table 2. There were no significant differences as a function of age group except for the question “Could this current headset replace a PACS workstation?” in which the 20–30 age group had more strongly agree ratings.

**Table 2:** Distributions of responses for post-use surveys on questions (1–5 scale where 1 = low, 5 = high)

	1	2	3	4	5	X^2^ & *p*-value	Medians	Means
ease of use	0%	0%	32%	36%	32%	X^2^ = 4.87 p = 0.088	4.0	4.0
overall experience	0%	0%	4%	52%	44%	X^2^ = 1.51 p = 0.471	4.0	4.4
ergonomics	0%	8%	24%	40%	28%	X^2^ = 1.36 p = 0.716	4.0	3.88
preference for diagnostic work	0%	16%	48%	20%	16%	X^2^ = 4.17 p = 0.244	3.0	3.36
preference for anatomic/diagnostic education	32%	16%	20%	24%	8%	X^2^ = 2.43 p = 0.657	3.0	2.6
AR headset for education	8%	16%	48%	28%	0%	X^2^ = 0.72 p = 0.870	3.0	2.96
performance Apple Vision Pro	0%	0%	20%	36%	44%	X^2^ = 1.99 p = 0.369	4.0	4.24
processing speed	0%	0%	8%	36%	56%	X^2^ = 5.12 p = 0.077	5.0	4.48
image quality	0%	4%	12%	56%	28%	X^2^ = 6.40 p = 0.094	4.0	4.08
image contrast	0%	0%	20%	28%	52%	X^2^ = 5.31 p = 0.070	5.0	4.32
image resolution	0%	4%	12%	40%	44%	X^2^ = 6.42 p = 0.093	4.0	4.24
viewing lighting	0%	0%	4%	36%	60%	X^2^ = 2.62 p = 0.269	5.0	4.56
scrolling	0%	4%	36%	24%	36%	X^2^ = 4.98 p = 0.173	4.0	3.92
window/level	0%	16%	28%	36%	20%	X^2^ = 2.15 p = 0.542	4.0	3.6
comparing studies	0%	12.5%	37.5%	20.83%	29.17%	X^2^ = 5.55 p = 0.136	3.5	3.67
dictation	0%	4%	32%	40%	24%	X^2^ = 1.24 p = 0.744	4.0	3.84
current version replace PACS workstation	12%	36%	28%	24%	0%	X^2^ = 8.02 p = 0.046	3.0	2.64
future version replace PACS workstation	0%	12%	24%	32%	32%	X^2^ = 4.57 p = 0.207	4.0	3.84
confidence improve pre to post	0%	8%	12%	64%	16%	X^2^ = 1.76 p = 0.623	4.0	3.88

According to the post-use survey, no one experienced nausea, but 12% experienced eye strain with all of them in the 30 + age group although it was not significant (X^2^ = 1.92, *p* = 0.166). Almost half (48%) of the respondents thought they could continuously use the device for 1–4 h in a shift, followed by < 1 h (28%), 5–8 h (12%), 8–12 h (8%), and < 30 min (4%) with no difference as a function of age group (X^2^ = 3.61, *p* = 0.462). In terms of the best modality for spatial computing (could select more than 1 response), 92% selected abdominal CT, 84% chest CT, 56% neuro MRI, 48% MSK MRI, 44% breast MRI, 44% abdominal MRI, 32% nuclear medicine, 28% image guided procedures, and 16% mammography with no difference as a function of age group (X^2^ = 1.20, *p* = 0.998).

## Discussion

This pilot study evaluated the perceived clinical/educational value of the Apple Vision Pro spatial computing platform within the radiology physician trainee environment. The AVP achieved high ratings for image quality, ease of use, and immersive interface, and participants were able to achieve device competency in under five minutes on average. These findings suggest that with minimal onboarding, users across many training and experience levels can effectively interact with the immersive imaging tools offered by spatial computing. The significant increase in perceived usefulness for multidisciplinary conferences further indicates that spatial computing may be especially valuable in collaborative review settings. Although only one survey domain reached statistical significance, nearly all domains trended positively, suggesting that with larger sample sizes and future work, additional applications may also show measurable improvements in perceived utility.

Our findings align with prior research highlighting the limitations of early-generation AR/VR devices in radiology [2, 18, 19]. However, prior studies using other headsets with lower-resolution displays cited issues with user fatigue, limited functionality, and lack of diagnostic-grade display fidelity. Micro-OLED display technology, like those used in the AVP, enables high luminance and deep contrast ratios, potentially improving the visibility of low-density findings such as fat stranding or mesenteric edema, which often appear in low-luminance regions [13]. At the time of this publication, the Apple Vision Pro is not an FDA-cleared Display Device for Diagnostic Radiology, cannot be calibrated to DICOM Grayscale Standard Display Function outlined in AAPM Report TG270, and the software applications used for medical image viewing include disclaimers that read “…not a medical device and must not be used for diagnosis” [20–22]. Therefore, no comparison was performed with PACS workstations, and statements regarding diagnostic performance are beyond the scope of this pilot.

Beyond the hardware, the AVP has a range of user-centric features that further enhance its potential utility. The headset supports intuitive navigation through gaze tracking and gesture control, allowing users to rotate, zoom, and manipulate imaging data in a natural and immersive way. These capabilities may help reduce cognitive load while enhancing spatial understanding; both of which are key elements for tasks such as preoperative planning, anatomical education, and reviewing complex cross-sectional medical imaging [12]. Additionally, integration with multimodal data sources like PACS, EHR, and 3D reconstruction software all processed locally on device allows for a unified and immersive workspace, supporting educational uses, and interdisciplinary collaboration [9]. Notably, the AVP’s connection to a broader software ecosystem provides access to a full suite of productivity and creative applications beyond healthcare, expanding its potential utility for users in both medical and non-medical contexts.

From an educational perspective, the AVP offers a compelling platform for modernizing radiology training through interactive image manipulation, 3D volume rendering, and integrated EHR access. Participants consistently highlighted the device’s high visual fidelity, responsive navigation, and immersive format, which enhanced their spatial understanding; an essential skill for interpreting cross-sectional anatomy. High ratings for processing speed, resolution, and scrolling accuracy further underscore its potential utility for medical imaging review. Notably, participants reported minimal discomfort, such as nausea or eye strain, contrasting with earlier-generation headsets. However, definitive conclusions about comfort after long term use remains limited as the total exposure time in this study was brief when considering a typical radiologist workday, which may reach 6–9 h of reading time [23]. While users still preferred conventional computer workstations for formal interpretation, the AVP’s high acceptance and minimal learning curve suggest it may serve as a valuable adjunct in academic and trainee-oriented settings.

This study had several limitations. The sample was small and drawn from a single academic institution, limiting the external validity of the findings. Only one attending physician participated, with the study cohort being predominantly trainees, reducing the ability to generalize findings to more experienced radiologists. The study design did not include objective performance metrics such as diagnostic accuracy, image interpretation speed, or fatigue indices. Moreover, participants used a simulated PACS environment, which may not fully represent real-world conditions or workload. Given the small, resident-heavy, single-center cohort, results should be interpreted as a pilot study rather than broadly generalizable findings. Importantly, this study was not designed or intended to claim that the AVP is ready for clinical use or would improve radiologic workflows. Rather, the goal was to assess whether users perceived any potential utility in the device and to identify which domains (i.e. education, visualization, or collaboration) may merit more rigorous, resource-intensive evaluation in future studies.

Future studies may focus on evaluating how spatial computing augmented learning compares to traditional teaching methods. For instance, learners may review three-dimensional anatomical concepts, such as the Circle of Willis, using conventional textbook-based instruction versus immersive 3D visualization in spatial computing. Clinical investigations may also include local institutional approval of these devices for experimental diagnostic use, enabling head-to-head comparison of spatial computing against conventional workstations as well as other AR/VR platforms. Such studies would be better suited to assess not only diagnostic accuracy and image quality, but also cognitive load, fatigue, and workflow efficiency during prolonged sessions.

In conclusion, this pilot study demonstrated that Apple Vision Pro with spatial computing was feasible, acceptable, and well tolerated for short educational usage in radiology physician training. Our findings pertain to usability and perceived educational value rather than diagnostic validation or evidence of workflow readiness. Future, adequately powered studies are needed to determine educational impact and to evaluate potential clinical integration of AVP in radiology.

## Supplementary Information

Below is the link to the electronic supplementary material.

Supplementary file1 (DOCX 500 KB)

## Data Availability

The data that support the findings of this study are available on request from the corresponding author.

## References

[1] Altun I, Turan O, Awan O. Revolutionizing radiology education: exploring innovative teaching methods. Abdom Radiol (NY) 2025; 10.1007/s00261-025-05010-x PMC12602628 40455218

[2] Lang M, Ghandour S, Rikard B, et al. Medical extended reality for radiology education and training. J Am Coll Radiol 2024;21(10):1583–1594. 10.1016/j.jacr.2024.05.006 38866067

[3] Pinsky BM, Panicker S, Chaudhary N, Gemmete JJ, Wilseck ZM, Lin L. The potential of 3D models and augmented reality in teaching cross-sectional radiology. Med Teach 2023;45(10):1108–1111. 10.1080/0142159X.2023.2242170 37542360

[4] Banerjee S, Pham T, Eastaway A, Auffermann WF, Quigley EP. The Use of Virtual Reality in Teaching Three-Dimensional Anatomy and Pathology on CT. J Digit Imaging 2023;36(3):1279–1284. 10.1007/s10278-023-00784-2 PMC9886418 36717519

[5] Weeks JK, Pakpoor J, Park BJ, et al. Harnessing Augmented Reality and CT to Teach First-Year Medical Students Head and Neck Anatomy. Acad Radiol 2021;28(6):871–876. 10.1016/j.acra.2020.07.008 PMC8011826 32828663

[6] Waisberg E, Ong J, Masalkhi M, et al. Apple Vision Pro and the advancement of medical education with extended reality. Can Med Educ J 2024;15(1):89–90. 10.36834/cmej.77634 PMC10961128 38528897

[7] Finos K, Datta S, Sedrakyan A, Milsom JW, Pua BB. Mixed reality in interventional radiology: a focus on first clinical use of XR90 augmented reality-based visualization and navigation platform. Expert Rev Med Devices 2024;21(8):679–688. 10.1080/17434440.2024.2379925 39054630

[8] Fidvi S, Holder J, Li H, Parnes GJ, Shamir SB, Wake N. Advanced 3D visualization and 3D printing in radiology. Adv Exp Med Biol 2023;1406:103–138. 10.1007/978-3-031-26462-7_6 37016113

[9] Uppot RN, Laguna B, McCarthy CJ, et al. Implementing virtual and augmented reality tools for radiology education and training, communication, and clinical care. Radiology 2019;291(3):570–580. 10.1148/radiol.2019182210 30990383

[10] Jung C, Wolff G, Wernly B, et al. Virtual and Augmented Reality in Cardiovascular Care: State-of-the-Art and Future Perspectives. JACC Cardiovasc Imaging 2022;15(3):519–532. 10.1016/j.jcmg.2021.08.017 34656478

[11] Masalkhi M, Waisberg E, Ong J, et al. Apple vision pro for ophthalmology and medicine. Ann Biomed Eng 2023;51(12):2643–2646. 10.1007/s10439-023-03283-1 37332003

[12] Rowe SP, Schneider R, Krueger S, Pryde V, Chu LC, Fishman EK. Augmented Reality With Cinematic Rendered 3-Dimensional Images From Volumetric Computed Tomography Data. J Comput Assist Tomogr 2023;47(1):67–70. 10.1097/RCT.0000000000001381 36194833

[13] Murphy PM, An JY, Wojdyla LM, et al. Diagnostic Performance of a Next-Generation Virtual/Augmented Reality Headset: A Pilot Study of Diverticulitis on CT. J Digit Imaging Inform med 2024; 10.1007/s10278-024-01292-7 PMC12572419 39495441

[14] Broderick RC, Spurzem GJ, Jeffery Reeves J, et al. First use of augmented reality headset in minimally invasive general surgery: seeing is believing. Surg Endosc 2025; 10.1007/s00464-025-11985-x PMC12408766 40640628

[15] Lan T, Liu S, Dai Y, Luo J, Han J, Jin Y. Real-time image fusion and Apple Vision Pro in laparoscopic microwave ablation of hepatic hemangiomas. NPJ Precis Oncol 2025;9(1):79. 10.1038/s41698-025-00867-z PMC11926265 40113974

[16] Armstrong DG, Bazikian S, Armstrong AA, Clerici G, Casini A, Pillai A. An augmented vision of our medical and surgical future, today? J Diabetes Sci Technol 2024;18(4):968–973. 10.1177/19322968241236458 PMC11307216 38439541

[17] Elsayed M, Kadom N, Ghobadi C, et al. Virtual and augmented reality: potential applications in radiology. Acta Radiol 2020;61(9):1258–1265. 10.1177/0284185119897362 31928346

[18] Beams R, Brown E, Cheng W-C, et al. Evaluation challenges for the application of extended reality devices in medicine. J Digit Imaging 2022;35(5):1409–1418. 10.1007/s10278-022-00622-x PMC9582055 35469355

[19] Pires F, Costa C, Dias P. On the use of virtual reality for medical imaging visualization. J Digit Imaging 2021;34(4):1034–1048. 10.1007/s10278-021-00480-z PMC8455774 34327628

[20] Bevins NB, Silosky MS, Badano A, Marsh RM, Flynn MJ, Walz-Flannigan AI. Practical application of AAPM Report 270 in display quality assurance: A report of Task Group 270. Med Phys 2020;47(9):e920–e928. 10.1002/mp.14227 32681556

[21] U.S. Food and Drug Administration. Health Center for Devices and Radiological Health [Internet]. Display Devices for Diagnostic Radiology 2022 [cited 2025 Oct 8]. Available from: https://www.fda.gov/regulatory-information/search-fda-guidance-documents/display-devices-diagnostic-radiology

[22] Visage Imaging. Visage on Apple - Visage Imaging [Internet]. 2025 [cited 2025 Oct 8]. Available from: https://visageimaging.com/platform/visage-on-apple/

[23] Krupinski EA, Berbaum KS, Caldwell RT, Schartz KM, Kim J. Long radiology workdays reduce detection and accommodation accuracy. J Am Coll Radiol 2010;7(9):698–704. 10.1016/j.jacr.2010.03.004 PMC2935843 20816631

