# Supplementary material for: Feasibility of Using the Apple Vision Pro for Diagnostic Radiology: User Experience and Perceived Clinical Utility
Source: J Imaging Inform Med. 2025 Nov 17;39(5):4808–16. doi: 10.1007/s10278-025-01745-7 (PMC13694668; doi:10.1007/s10278-025-01745-7)
Supplement: Supplementary file 1 [file 10278_2025_1745_MOESM1_ESM.docx]

**Appendix Legends:**

A.1- User Survey Form

A.2 - Research Lead Form
